# Socio-demographic differences in the dietary inflammatory index from National Health and Nutrition Examination Survey 2005–2018: a comparison of multiple imputation versus complete case analysis

**DOI:** 10.1017/S1368980024001800

**Published:** 2024-09-27

**Authors:** Rachel J Meadows, Electra D Paskett, Julie K Bower, Gail L Kaye, Stanley Lemeshow, Randall E Harris

**Affiliations:** 1 Center for Epidemiology & Healthcare Delivery Research, JPS Health Network, 1500 South Main Street, Fort Worth, TX 76104, USA; 2 Division of Health Services Management and Policy, College of Public Health, The Ohio State University, Columbus, OH, USA; 3 Division of Epidemiology, College of Public Health, The Ohio State University, Columbus, OH, USA; 4 College of Medicine, Comprehensive Cancer Center, The Ohio State University, Columbus, OH, USA; 5 Division of Health Behavior and Health Promotion, College of Public Health, The Ohio State University, Columbus, OH, USA; 6 Division of Biostatistics, College of Public Health, The Ohio State University, Columbus, OH, USA

**Keywords:** Dietary intake, Multiple imputation, Nutritional epidemiology, Disparities, NHANES

## Abstract

**Objective:**

Studies using the dietary inflammatory index often perform complete case analyses (CCA) to handle missing data, which may reduce the sample size and increase the risk of bias. Furthermore, population-level socio-economic differences in the energy-adjusted dietary inflammatory index (E-DII) have not been recently studied. Therefore, we aimed to describe socio-demographic differences in E-DII scores among American adults and compare the results using two statistical approaches for handling missing data, i.e. CCA and multiple imputation (MI).

**Design:**

Cross-sectional analysis. E-DII scores were computed using a 24-hour dietary recall. Linear regression was used to compare the E-DII scores by age, sex, race/ethnicity, education and income using both CCA and MI.

**Setting:**

USA.

**Participants:**

This study included 34 547 non-Hispanic White, non-Hispanic Black and Hispanic adults aged ≥ 20 years from the 2005–2018 National Health and Nutrition Examination Survey.

**Results:**

The MI and CCA subpopulations comprised 34 547 and 23 955 participants, respectively. Overall, 57 % of the American adults reported 24-hour dietary intakes associated with inflammation. Both methods showed similar patterns wherein 24-hour dietary intakes associated with high inflammation were commonly reported among males, younger adults, non-Hispanic Black adults and those with lower education or income. Differences in point estimates between CCA and MI were mostly modest at ≤ 20 %.

**Conclusions:**

The two approaches for handling missing data produced comparable point estimates and 95 % CI. Differences in the E-DII scores by age, sex, race/ethnicity, education and income suggest that socio-economic disparities in health may be partially explained by the inflammatory potential of diet.

Chronic inflammation contributes to the initiation and progression of numerous chronic health conditions that differentially burden populations based on demographic and socio-economic factors^(1)^. Adverse social determinants of health can upregulate systemic inflammation from childhood through adulthood and lead to the early development of chronic diseases^(1,2)^. Nonetheless, dietary change is a promising strategy to modify chronic inflammation and thereby reduce chronic disease risks^(3)^. Dietary intake is often studied using dietary indices such as the Healthy Eating Index, Dietary Approaches to Stop Hypertension and Dietary Guidelines for Americans^(4,5)^. Substantial socio-economic and racial/ethnic differences in dietary behaviours have been long observed using these traditional dietary indices; however, these indices were not developed to directly measure the pro- or anti-inflammatory effects of diets. Dietary inflammation may be a key contributor to the socio-economic disparities observed in various chronic health conditions.

The dietary inflammatory index (DII) is a novel dietary assessment tool developed to directly measure the inflammatory potential of the overall diet^(6,7)^. The DII characterises the overall inflammatory potential of a diet using up to forty-five dietary components and has been validated with various markers of inflammation in several populations^(8,9)^. The DII correlates moderately (*r* = 0·52–0·65) with traditional dietary indices (i.e. the Healthy Eating Index and Dietary Approaches to Stop Hypertension)^(10)^. The DII is sometimes adjusted for overall energy intake, referred to as the energy-adjusted DII (E-DII)^(9)^, which assesses inflammatory potential per 4184 kJ (1000 kcal) of energy intake. The energy-adjusted version is critical for evaluating the effect of the inflammatory potential, independent of the overall energetic intake. To date, over 1200 studies using the DII/E-DII have been published; however, these studies are often limited by not examining missing data mechanisms, resulting in inappropriate handling of missing data, which can potentially result in bias and weaken the validity of the study conclusions^(7,11–13)^.

Missing data mechanisms are often categorised into the following three types: missing completely at random (MCAR), missing at random (MAR) and missing not at random (MNAR)^(14)^. MCAR occurs when the probability of missing data is the same for each participant; for example, if a weighing scale breaks, some participants have missing BMI data. The missing data are unrelated to the observed or unobserved data. MCAR is the strongest assumption and is unrealistic in typical epidemiologic studies^(15)^. When missing data are MCAR, complete case analysis (CCA) can be valid. Unlike MCAR, MAR occurs when missing data are associated with other collected data. For example, if missing data on alcohol intake were more common in younger age groups than in the older age groups, then the missing alcohol data would be MAR. When missing data are MAR, multiple imputation (MI) methods are recommended^(13,16,17)^. Lastly, MNAR occurs when missing data depend on unobserved data, such as the variable itself. For example, if the missing data on sugar intake were more common among those who consume high levels of sugar than among others, regardless of age, sex or other observed variables, then the missing data on sugar intake would be MNAR. Because MNAR data depend on unobserved or unknown information, stronger assumptions are required. A common approach is to conduct sensitivity analyses wherein assumptions are varied and result in several possible conclusions^(15,18)^.

Despite strong evidence demonstrating the importance and implementation of statistical methods for the appropriate analysis of missing data, the most basic and commonly used approach, CCA, continues to be used in epidemiologic studies, including dietary research^(12,19,20)^. Using partial data that include only participants with complete data can exclude information that may be vital to studying disparities between subgroups. Because MAR, rather than MCAR or MNAR, is commonly observed in epidemiologic studies^(13)^, this study focussed on comparing the basic approach of CCA with a more appropriate approach, i.e. MI^(17)^. This study aimed (1) to investigate differences in E-DII scores by age, sex, race/ethnicity, education and income and (2) to compare the results using two statistical approaches for handling missing data, i.e. CCA and MI.

## Methods

### Study population

This study was a secondary data analysis of non-Hispanic White (NHW), non-Hispanic Black (NHB) and Hispanic adults aged ≥ 20 years, who participated in the National Health and Nutrition Examination Survey (NHANES) 2005–2018 cycles. The NHANES program includes interviews, physical examinations and laboratory tests. The NHANES uses a clustered design with multistage probability sampling to obtain a study sample representative of the civilian, non-institutionalised U.S. population. Detailed data collection methods for the NHANES are available on the NHANES website^(21)^.

### Measures

#### Questionnaire data

##### Demographic information

Self-reported information on sex (male or female), age, marital status (married, widowed, divorced, separated, never married or living with a partner), race/ethnicity (NHW, NHB or Hispanic including Mexican Americans and other Hispanics) and education level (less than 9th grade, 9–11th grade, high school graduate or equivalent, some college or associates degree or college graduate or above) was collected. Income was used to calculate the family income-to-poverty ratio (FIPR) based on the U.S. Department of Health and Human Services poverty guidelines for each year. FIPR was categorised based on eligibility levels for insurance subsidies under the Patient Protection and Affordable Care Act as follows: high income (FIPR ≥ 4), middle income (FIPR > 1 and < 4) and at or below the federal poverty level (FIPR ≤ 1)^(22)^.

##### Dietary intake

The dietary assessment component of NHANES, ‘What We Eat in America’, is a joint programme between the U.S. Department of Health and Human Services and the U.S. Department of Agriculture. Dietary data were collected via a single 24-hour recall at the NHANES mobile examination centres. Interviewers used the U.S. Department of Agriculture’s Automated Multiple-Pass Method, a 5-step interview process, to record the dietary intakes efficiently and accurately^(21,23)^. The U.S. Department of Agriculture’s Food Surveys Research Group then used the 24-hour recall to generate nutrient files for each participant^(24)^. The ‘What We Eat in America’ data were used to estimate the prevalence of nutrient adequacy for foods, food groups, nutrients and dietary patterns and to inform nutrition programs and policies for the U.S. population^(25)^.

For our study, nutrient files were used to calculate the DII scores; full details of the process have been described previously^(6)^. Briefly, the DII comprises forty-five food parameters, including individual nutrients (e.g. *n*-3 fatty acids), compounds (e.g. flavonoids), foods (e.g. garlic) and drinks (e.g. alcohol), which are associated with an inflammatory effect score. The forty-five food parameters and inflammatory effect scores were based on a review of 1943 published articles, and they were scored on their associations with the following inflammatory biomarkers: C-reactive protein, IL-1*β*, IL-4, IL-6, IL-10 and TNF-*α* ^(6)^. The E-DII scores were calculated per 4184 kJ (1000 kcal) of food consumed to control for the effect of total energy intake, using the energy-standardised version of the global database. The theoretical range of possible values is from −9 to 8, where 0 represents a neutral diet, a score < 0 represents an anti-inflammatory potential of the diet, and a score > 0 represents a pro-inflammatory potential of the diet^(6)^. Nonetheless, studies often report ranges between −5 and +5^(11,26)^. The E-DII has been validated in association with inflammatory biomarkers in several population-based studies^(9,27)^.

The following twenty-eight of the forty-five E-DII parameters were available in the NHANES for the calculation of the overall E-DII scores: total energy, carbohydrate, protein, total fat, SFA, MUFA, PUFA, alcohol, fibre, cholesterol, niacin (vitamin B_3_), riboflavin (vitamin B_2_), thiamin (vitamin B_1_), Fe, Mg, Zn, Se, vitamin A, vitamin B_6_, vitamin B_12_, vitamin C, vitamin D, vitamin E, folic acid, beta carotene, *n*-6, *n*-3 and caffeine^(9)^. Total energy intake was used to standardise the nutrient intake per 4184 kJ (1000 kcal) consumed. The remaining seventeen food parameters were not collected in the NHANES or were not collected consistently across all included years (trans-fat, anthocyanidins, eugenol, flavan-3-ol, flavones, flavonols, flavanones, isoflavones, garlic, ginger, onion, saffron, turmeric, pepper, thyme/oregano, rosemary and green/black tea). Prior studies have shown no significant change in the predictive ability when at least twenty-eight parameters are used compared with the full list of forty-five^(8)^. Published studies include twenty-seven food parameters on average, mainly because of the frequently limited representation of dietary information in structured questionnaires^(11,28)^.

##### Chronic health conditions

Major chronic health conditions associated with chronic inflammation were self-reported individually by condition^(29)^. Indicator variables were created for each of the following conditions: diabetes, cancer, stroke, heart attack, angina, coronary heart disease, congestive heart failure, arthritis, liver disease, asthma, bronchitis and emphysema. Depressive symptoms were assessed using the nine-question Patient Health Questionnaire, employing a cut-off score ≥ 10, which indicates moderate to severe depressive symptoms^(30)^.

##### Family history of diabetes, heart attack or angina

Participants were asked about their family history (father, mother, sisters or brothers only) for diabetes and heart attack or angina before the age of 50.

##### Food security

Ten questions from the U.S. Department of Agriculture Adult Food Security Module enquired about adult food insecurity in the last 12 months (e.g. scarcity of food, inability to afford to buy balanced meals and cutting size of meals or skipping meals due to lack of money)^(31)^. The NHANES data categorises responses into the following categories: full food security (no affirmative responses), marginal food security (1–2 affirmative responses), low food security (3–5 affirmative responses) and very low food security (6–10 affirmative responses)^(32)^. We merged low food security and very low food security into a single category to maintain sufficient cell size for analyses.

##### Lifestyle behaviours

Sleep: The participants were asked, ‘How much sleep do you usually get at night on weekdays or workdays?’ Sleep was categorised as short sleep (≤ 6 h/night), normal sleep (7–9 h/night) and long sleep (≥ 10 h).

Smoking: Smoking status was categorised as never smoker (< 100 cigarettes smoked in their lifetime but not currently smoking cigarettes), current smoker (≥ 100 cigarettes smoked in their lifetime and currently smoking cigarettes) or former smoker (≥ 100 cigarettes smoked in their lifetime but not currently smoking cigarettes)^(33)^.

Physical activity: The Global Physical Activity Questionnaire was used to assess daily minutes of moderate- and vigorous-intensity physical activity for transportation (walking or biking), household or yard work and recreation^(34)^. The metabolic equivalents per week were calculated, and the participants were categorised as ‘inactive’ (no reported physical activity), ‘somewhat active’ (> 0 to < 500 metabolic equivalents) and ‘active’ (meeting physical activity guidelines of ≥ 500 metabolic equivalents per week)^(35)^.

#### Examination data

##### BMI

Trained health technicians measured participants’ height and weight. BMI was calculated as weight in kilograms divided by height in meters squared and categorised as follows: underweight (< 18·5 kg/m^2^), healthy weight (18·5–24·9 kg/m^2^), overweight (25·0–29·9 kg/m^2^) and obesity (≥ 30·0 kg/m^2^)^(36)^.

##### Hypertension

Hypertension was defined as an average systolic blood pressure ≥ 140 mmHg, diastolic blood pressure ≥ 90 mmHg or self-reported use of antihypertensive medications.

##### Hypercholesterolaemia

Hypercholesterolaemia was defined as total serum cholesterol ≥ 240 mg/dl or self-reported use of cholesterol-lowering medications.

### Analyses

#### Descriptive statistics

**Fig. 1:**
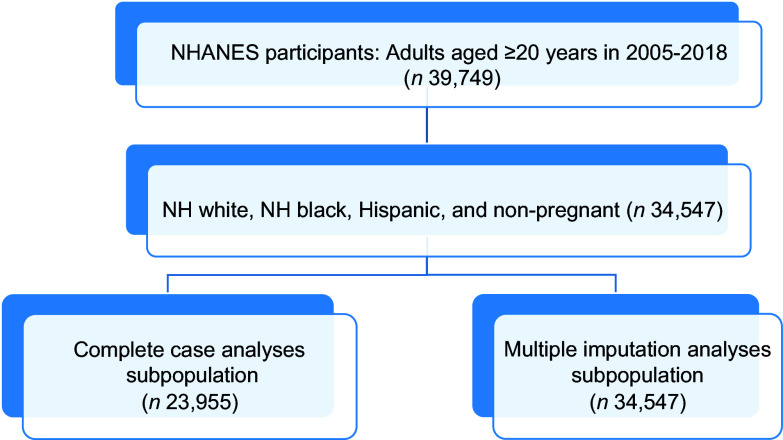
Selection of participants for complete case analyses and multiple imputation subpopulations from National Health and Nutrition Examination Survey years 2005–2018. The complete case analyses subpopulation included only participants with no missing data on exposure, outcome or covariates of interest. The multiple imputation analyses subpopulation included all eligible participants, regardless of missing data on exposure, outcome or covariates of interest. NHANES, National Health and Nutrition Examination Survey; NH, non-Hispanic.

We calculated the weighted mean and proportions to describe the characteristics of the overall study population based on non-missing data^(37)^. We used box plots to report medians and interquartile ranges and graphically present the E-DII scores across the NHANES years (Fig. 1).

#### Complete case analyses and multiple imputation subpopulations

##### Complete case analyses subpopulation

This subpopulation was restricted to eligible participants with complete data for all exposure, outcome and covariate variables.

##### Multiple imputation subpopulation

This subpopulation included all eligible participants, regardless of missing data on exposure, outcome or covariates. Missing data for all the variables were imputed as described below.

#### Multiple imputation approach

MI is a flexible and commonly used approach that can accommodate variables with up to 50 % missing data^(13,17)^ to help minimise bias in national surveys^(38,39)^. We used MI by chained equations with the incorporation of NHANES sample weights to impute all missing data for variables stratified by sex (Table 1)^(38)^. The imputed variables included socio-demographic characteristics, family history, chronic conditions, lifestyle behaviours and E-DII scores. Missing E-DII values indicated adults who did not participate in the 24-hour dietary recall, and an overall E-DII score was imputed (i.e. individual E-DII parameters were not imputed). Among participants who reported a 24-hour dietary recall, variation in the number of food parameters was possible (e.g. Participant A reported a food intake that included 23 E-DII parameters, while Participant B reported a food intake that included twenty-seven E-DII parameters), but individual ‘missing’ E-DII parameters were not imputed. Notably, ‘missing’ individual E-DII parameters indicate that the participant did not report food intake that included that particular E-DII parameter; hence, it would not contribute to their overall E-DII score. Overall, each study variable had < 10 % missing information.

**Table 1:** Characteristics of American Non-Hispanic White, Non-Hispanic Black and Hispanic adults aged ≥ 20 years from the National Health and Nutrition Examination Survey 2005–2018 (*n* 34 547)

Characteristic	%	Weighted Mean	se	Missing*
Age (years)		47·83	0·23	0 %
Female	51·28 %			0 %
Race/ethnicity				0 %
Non-Hispanic White	72·51 %			
Non-Hispanic Black	12·31 %			
Hispanic	15·17 %			
Family income-to-poverty ratio				7·51 %
High	33·89 %			
Middle	45·29 %			
Low	13·31 %			
Education level				0 %
College graduate or above	27·99 %			
Some college or associates degree	31·51 %			
High school/equivalent	23·89 %			
Less than high school	16·50 %			
Married/living with partner	62·51 %			<1 %
BMI				4·75 %
Underweight (< 18·5 kg/m^2^)	1·46 %			
Healthy weight (18·5–24·9 kg/m^2^)	25·87 %			
Overweight (25·0–29·9 kg/m^2^)	31·45 %			
Obese (≥ 30·0 kg/m^2^)	36·47 %			
Family history of diabetes	38·47 %			1·81 %
Family history of heart disease	13·07 %			2·28 %
Hypertension	38·03 %			5·19 %
Hypercholesterolaemia	9·67 %			1·27 %
≥ 1 chronic disease	48·69 %			<1 %
Low or very low food security	13·73 %			1·68 %
Diet				9·11 %
E-DII score		0·44	0·026	
Pro-inflammatory diet	56·85 %			
Anti-inflammatory diet	34·05 %			
Meets physical activity recommendations	45·29 %			< 1 %
Current smoker	20·72 %			< 1 %
Short sleep	30·11 %			3·17 %

E-DII, energy-adjusted dietary inflammatory index.

* Includes non-responses and ‘Don’t know’ or ‘Refuse’ responses.

In this study, we generated fifteen imputed datasets, each containing a set of plausible replacement values for missing data points. Multiple imputed datasets were analysed, and overall estimates were obtained by averaging the estimates from each individually imputed dataset. The estimated standard errors and confidence intervals include an imputation variance component^(13,16,17,38,39)^.

#### Linear regression models

Linear regression models were used to estimate the association between sex and E-DII scores. We used sex-stratified models to assess independent associations between the 5-year age groups, race/ethnicity, education, FIP and E-DII scores. All models were run using the CCA and MI subpopulations to facilitate comparison. The absolute value of the percent change of *β* was calculated to compare coefficients between the CCA and MI subpopulation estimates for unadjusted and adjusted models. The percent change formula was as follows: | (CCA *β* – MI *β*)/CCA *β* | × 100.

We considered the following potential confounders: NHANES survey year, dietary intake day of the week, age, marital status, BMI, food security, family history of diabetes, family history of heart disease, chronic health conditions, physical activity, sleep and smoking status. In models with age as the main predictor, we categorised age into 5-year groups; in models with age as a covariate, age was modelled as a continuous variable (justified using fractional polynomials). The final models included significant confounders defined by the 10 % change-in-estimate criterion^(40)^. We also tested whether differences in the unadjusted E-DII scores by sex and race/ethnicity differed by the NHANES year. All analyses were conducted using STATA version 16 (StataCorp LLC.)

We confirmed several assumptions of linear regression for the final models. Scatter plots were used to assess the linearity of the associations between exposure and outcome variables. Histograms were used to assess the distribution of the residual normality. The regression assumptions of homogeneity of variance and collinearity were verified using a visual inspection of residual *v*. predicted value plots and a variance inflation factor of < 5, respectively. The NHANES survey weights were incorporated into all analyses and were robust to heteroskedasticity, clustering, non-response errors and complex probability sampling designs^(21)^.

## Results

Based on non-missing data, Table 1 presents sample characteristics of the 34 547 NHW, NHB and Hispanic adults aged ≥ 20 years, who participated in the NHANES cycles 2005–2018. Among the included participants, 51 % were female and 49 % were male, with a mean age of 48 years. Furthermore, 73 % were NHW, 12 % were NHB and 15 % were Hispanic. In total, 36 % of the participants had an obese BMI, 31 % had an overweight BMI and 49 % reported at least one chronic health condition. Less than half (45 %) met the physical activity recommendations. Table 1 describes the missing data for each variable imputed in the regression models.

The MI and CCA subpopulations comprised 34 547 and 23 955 participants, respectively. Sex and BMI did not differ between the subpopulations (*P* = 0·373 and *P* = 0·24, respectively). Age (*P* < 0·001), race/ethnicity (*P* < 0·001), education (*P* < 0·01) and food security (*P* = 0·004) differed between the subpopulations although absolute differences were minimal. The CCA subpopulation had a slightly lower mean age (47·42 years *v*. 47·83 years), more NHW participants (74·8 % *v*. 72·5 %), more college graduates (29·46 % *v*. 28·02 %) and more individuals with full food security (77·47 % *v*. 76·79 %) compared with the MI subpopulation.

**Fig. 2:**
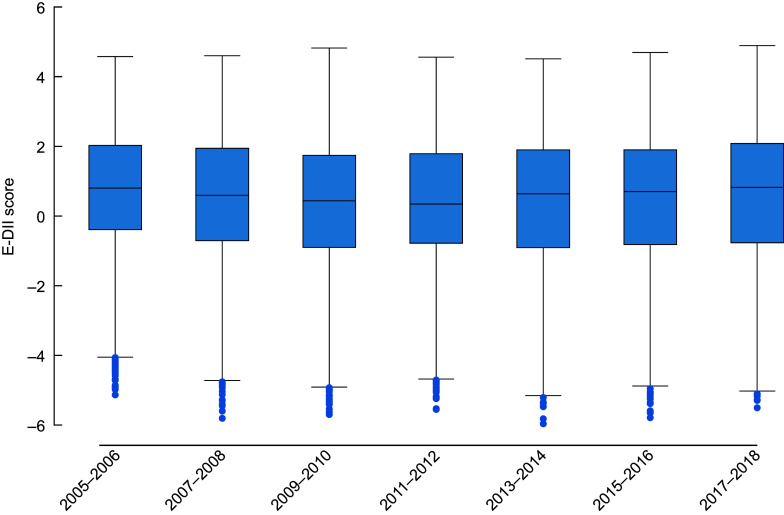
Box plots of energy-adjusted dietary inflammatory index scores by two-year National Health and Nutrition Examination Survey cycles. The range of observed E-DII scores was -5·96 (anti-inflammatory dietary potential) to 4·90 (pro-inflammatory dietary potential). (Bars represent the interquartile range; dots represent outliers.) E-DII, energy-adjusted dietary inflammatory index.

Overall, 57 % of adults reported E-DII scores indicative of a pro-inflammatory dietary potential, while only 34 % reported scores indicative of an anti-inflammatory dietary potential. The E-DII scores in this sample ranged from –5·96 (anti-inflammatory dietary potential) to a maximum score of 4·90 (pro-inflammatory dietary potential), and the overall mean score was pro-inflammatory (0·44 (95 % CI: 0·39, 0·49)). The average E-DII scores changed slightly over time, decreasing from 2005 to 2012 and increasing from 2012 to 2018 (Fig. 2). Nonetheless, the magnitude of temporal changes in the E-DII score was minimal (*β* = –0·025 (95 % CI: –0·053, 0·0020).

### Unadjusted associations

Similar patterns were observed in the CCA and MI subpopulations for all unadjusted comparisons of E-DII scores by sex, age, race/ethnicity, education and FIPR (Tables 2 and 3). Mean E-DII scores indicated greater anti-inflammatory potential of diet among women (*β* = 0·24 (95 % CI: 0·17, 0·30)) than among men (*β* = 0·66 (95 % CI: 0·60, 0·71)). Regression models using CCA and MI reflected similar patterns in the E-DII scores among women compared with men (CCA: *β* = –0·43 (95 % CI: –0·49, –0·38); MI: *β* = –0·42 (95 % CI: –0·47, –0·37); Table 2). The E-DII scores indicated a trend towards greater anti-inflammatory dietary potential with increasing age among both males and females. NHB adults had the highest average E-DII scores (*β* = 0·84 (95 % CI: 0·78, 0·90)), NHW adults had intermediate average scores (*β* = 0·39 (95 % CI: 0·33, 0·46)) and Hispanic adults had the lowest average scores (*β* = 0·35 (95 % CI: 0·30, 0·40)). The E-DII scores indicated a greater anti-inflammatory dietary potential with higher levels of education and FIPR. Differences in unadjusted point estimates between the CCA and MI subpopulations were mostly modest at ≤ 20 %.

**Table 2:** Unadjusted and adjusted linear regression for the associations between sex and energy-adjusted dietary inflammatory index scores

	CCA (*n* 23 955)		MI (*n* 34 547)		|%| change*
	*β*	95 % CI	*β*	95 % CI	
	Unadjusted association				
Male	Referent		Referent		
Female	–0·43	–0·49, –0·38	–0·42	–0·47, –0·37	2 %
	Adjusted association†				
Male	Referent		Referent		
Female	–0·41	–0·46, –0·36	–0·41	–0·46, –0·36	0 %

E-DII, energy-adjusted dietary inflammatory index; CCA, complete case analyses; MI, multiple imputation.

* Absolute value for the percent change in point estimates between the CCA and MI models.

† Adjusted for the following covariates: National Health and Nutrition Examination Survey year, dietary intake day of the week, BMI, food insecurity, family history of heart disease, family history of diabetes, comorbidities, smoking, physical activity and sleep.

**Table 3:** Unadjusted linear regression for the associations between socio-demographic factors and energy-adjusted dietary inflammatory index scores stratified by sex

	Males					Females				
	CCA (*n* 11 925)		MI (*n* 17 031)			CCA (*n* 12 030)		MI (*n* 17 516)		
	*β*	95 % CI	*β*	95 % CI	|%| change*	*β*	95 % CI	*β*	95 % CI	|%| change*
Age groups (years)										
20–24	Referent		Referent			Referent		Referent		
25–29	–0·15	–0·31, 0·01	–0·12	–0·27, 0·03	20 %	–0·33	–0·56, –0·10	–0·23	–0·45, –0·02	30 %
30–34	–0·29	–0·48, –0·10	–0·24	–0·41, –0·07	17 %	–0·43	–0·66, 0·21	–0·37	–0·57, –0·17	14 %
35–39	–0·30	–0·47, –0·18	–0·24	–0·41, –0·08	20 %	–0·42	–0·63, –0·21	–0·46	–0·67, –0·25	10 %
40–44	–0·34	–0·55, –0·12	–0·27	–0·46, –0·07	18 %	–0·41	–0·63, –0·19	–0·40	–0·60, –0·19	2 %
45–49	–0·45	–0·65, –0·26	–0·38	–0·56, –0·21	16 %	–0·51	–0·74, –0·29	–0·44	–0·65, –0·23	14 %
50–54	–0·44	–0·61, –0·27	–0·43	–0·60, –0·26	2 %	–0·75	–1·02, –0·49	–0·70	–0·94, –0·45	7 %
55–59	–0·60	–0·82, –0·39	–0·55	–0·75, –0·36	8 %	–0·87	–1·11, 0·63	–0·84	–1·05, –0·63	3 %
60–64	–0·68	−0·90, 0·47	–0·60	–0·78, –0·42	12 %	–0·97	–1·21, –0·73	–0·94	–1·17, –0·72	3 %
65–69	–0·92	–1·11, –0·75	–0·90	–1·09, –0·70	2 %	–1·06	–1·34, –0·78	–1·03	–1·28, –0·78	3 %
70–74	–1·18	1·40, 0·96	–1·07	–1·27, –0·86	9 %	–1·26	–1·50, –1·02	–1·17	–1·39, –0·96	7 %
≥ 75	–1·22	–1·42, –1·03	–1·13	–1·30, –0·96	7 %	–1·25	–1·46, –1·03	–1·12	–1·31, –0·94	10 %
Race/ethnicity										
NHW	Referent		Referent			Referent		Referent		
NHB	0·38	0·28, 0·47	0·31	0·23, 0·40	18 %	0·65	0·52, 0·77	0·54	0·44, 0·64	17 %
Hispanic	–0·10	–0·20, 0·002	–0·10	–0·19, 00·02	0 %	0·049	–0·06, 0·16	0·0043	–0·09, 0·10	91 %
Education level										
College	Referent		Referent			Referent		Referent		
Some college or associates degree	0·70	0·57, 0·84	0·64	0·51, 0·77	9 %	0·75	0·61, 0·89	0·73	0·61, 0·85	3 %
High school/ equivalent	0·93	0·79, 1·05	0·84	0·72, 0·96	10 %	1·00	0·86, 1·14	0·95	0·82, 1·08	5 %
Less than high school	0·86	0·74, 0·99	0·73	0·61, 0·84	15 %	0·93	0·76, 1·10	0·80	0·66, 0·94	14 %
FIPR										
High	Referent		Referent			Referent		Referent		
Middle	0·43	0·33, 0·53	0·41	0·32, 0·50	5 %	0·46	0·35, 0·57	0·46	0·36, 0·56	0 %
Low	0·63	0·51, 0·75	0·57	0·46, 0·69	10 %	0·86	0·73, 0·99	0·74	0·63, 0·86	14 %

CCA, complete case analyses; MI, multiple imputation; NHW, non-Hispanic White; NHB, non-Hispanic Black; FIPR, family income-to-poverty ratio.

* Absolute value for the percent change in point estimates between the CCA and MI models.

### Adjusted associations

In the adjusted models (Table 4), all patterns of E-DII scores by sex, age, race/ethnicity, education and FIPR remained. Similar patterns were observed in the CCA and MI subpopulations for all adjusted comparisons. Differences in adjusted point estimates between the CCA and MI subpopulations were mostly modest at ≤ 20 %.

**Table 4:** Adjusted linear regression for the associations between socio-demographic factors and energy-adjusted dietary inflammatory index scores stratified by sex

	Males					Females				
	CCA (*n* 11 925)		MI (*n* 17 031)			CCA (*n* 12 030)		MI (*n* 17 516)		
	*β*	95 % CI	*β*	95 % CI	|%| change*	*β*	95 % CI	*β*	95 % CI	|%| change*
Age groups (years)†										
20–24	Referent		Referent			Referent		Referent		
25–29	–0·15	–0·30, 0·01	–0·16	–0·30, –0·01	7 %	–0·35	–0·54, –0·15	–0·28	–0·48, –0·08	20 %
30–34	–0·35	–0·52, –0·17	–0·31	–0·48, –0·15	11 %	–0·50	–0·70, –0·31	–0·46	–0·64, –0·28	8 %
35–39	–0·36	–0·53, –0·20	–0·31	–0·48, –0·15	14 %	–0·55	–0·73, –0·37	–0·58	–0·76, –0·39	5 %
40–44	–0·43	–0·64, –0·23	–0·36	–0·56, –0·16	16 %	–0·55	–0·74, –0·37	–0·54	–0·72, –0·36	2 %
45–49	–0·51	–0·69, –0·32	–0·44	–0·61, –0·27	14 %	–0·68	–0·89, –0·47	–0·60	–0·81, –0·40	12 %
50–54	–0·53	–0·69, –0·38	–0·53	–0·69, –0·36	0 %	–0·95	–1·19, –0·71	–0·88	–1·12, –0·65	7 %
55–59	–0·70	–0·90, –0·49	–0·67	–0·86, –0·48	4 %	–1·09	–1·31, –0·86	–1·05	–1·25, –0·85	4 %
60–64	–0·77	–0·96, –0·57	–0·70	–1·20, –0·79	9 %	–1·21	–1·45, –0·97	–1·21	–1·43, –0·99	0 %
65–69	–0·97	–1·16, –0·78	–1·00	–1·38, –0·95	3 %	–1·21	–1·49, –0·94	–1·19	–1·46, –0·93	2 %
70–74	–1·22	–1·44, –1·00	–1·17	–1·38, –0·95	4 %	–1·51	–1·72, –1·29	–1·42	–1·63, –1·22	6 %
≥ 75	–1·32	–1·52, –1·12	–1·32	–1·49, –1·15	0 %	–1·48	–1·69, –1·26	–1·40	–1·60, –1·20	5 %
Race/ethnicity‡										
NHW	Referent		Referent			Referent		Referent		
NHB	0·18	0·090, 0·26	0·16	0·073, 0·25	11 %	0·28	0·18, 0·39	0·27	0·17, 0·36	4 %
Hispanic	–0·40	–0·49, –0·30	–0·41	–0·50, –0·32	3 %	–0·22	–0·33, –0·11	–0·23	–0·33, –0·13	5 %
Education level§										
College	Referent		Referent			Referent		Referent		
Some college or associates degree	0·49	0·35, 0·63	0·45	0·31, 0·58	8 %	0·46	0·32, 0·59	0·45	0·33, 0·57	2 %
High school/ equivalent	0·66	0·53, 0·80	0·61	0·48, 0·73	8 %	0·68	0·56, 0·81	0·67	0·56, 0·79	1 %
Less than high school	0·63	0·50, 0·77	0·58	0·46, 0·71	8 %	0·57	0·39, 0·74	0·56	0·42, 0·71	2 %
FIPR§										
High	Referent		Referent			Referent		Referent		
Middle	0·28	0·18, 0·38	0·29	0·20, 0·39	4 %	0·18	0·070, 0·29	0·20	0·10, 0·30	11 %
Low	0·34	0·21, 0·47	0·34	0·22, 0·47	0 %	0·22	0·090, 0·34	0·17	0·049, 0·30	23 %

CCA, complete case analyses; MI, multiple imputation; NHW, non-Hispanic White; NHB, non-Hispanic Black; FIPR, family income-to-poverty ratio.

All models are adjusted for the following covariates: National Health and Nutrition Examination Survey year, dietary intake day of the week, BMI, food security, family history of heart disease, family history of diabetes, comorbidities, smoking, physical activity and sleep.

* Absolute value for the percent change in point estimates between the CCA and MI models.

† Adjusted for the additional covariates: race/ethnicity and family income-to-poverty ratio.

‡ Adjusted for the additional covariates: age and family income-to-poverty ratio.

§ Adjusted for the additional covariates: age and race/ethnicity.

## Discussion

This study estimated the differences in E-DII scores by sex, age, race/ethnicity, education and FIPR among a U.S. population-based sample of 34 547 NHW, NHB and Hispanic adults, using two different approaches for handling missing data. The CCA and MI approaches resulted in comparable associations between E-DII scores and sex, age, race/ethnicity, education and FIPR. The consistency of point estimates between CCA and MI supports the robustness of MI in handling missing data. The MI approach facilitated the inclusion of an additional 44 % of participants who were ineligible for CCA due to missing data. Overall, 57 % of American adults reported E-DII scores associated with a high potential for supporting pro-inflammatory processes. Anti-inflammatory E-DII scores were associated with increasing age. NHB adults and adults with low education levels and FIPR reported the highest intakes of diets with pro-inflammatory potential.

Our results should be interpreted while considering several limitations. First, diet was assessed using a single 24-hour recall, which may be subject to various biases (e.g. recall and social desirability bias) and does not fully capture long-term dietary intake or patterns. Nonetheless, the use of the automated multiple-pass method reduces reporting bias, and results from dietary studies using the NHANES are often consistent with those of studies that use more robust dietary intake measures^(23,41)^. Additionally, the E-DII as a measure to assess dietary inflammation has some limitations. For instance, many food components may have U-shaped associations with health outcomes that are not fully accounted for in the E-DII, such as vitamins A and E and alcohol. Finally, the crude categorisations of sex, race/ethnicity and socio-economic status in this study may not have fully captured the true complexity of these factors. For example, binary sex and adult socio-economic status capture only a portion of the true range that can influence dietary behaviours. Nonetheless, the results of this study point to methodological improvements and call attention to subgroup differences, which are critical for assessing the impact of dietary behaviours on health outcomes.

Our study demonstrates the advantage of MI over CCA, which is critical for maintaining the national representativeness of the NHANES data. Many prior DII/E-DII studies using national-level data have used CCA, which limited the sample size by at least 20 %^(7,11,26)^. Despite the similar associations between CCA and MI in this study, CCA is not guaranteed to be comparable across all associations of interest. The risk of bias from CCA may be higher depending on the outcomes of interest or missing data mechanisms. The reduced sample size also limits the ability to examine potential differences by subgroups or effect measure modifiers, which may be critical when studying dietary behaviour and health outcomes. Others have discussed the advantages of MI in producing unbiased and valid estimates of associations; however, MI has not been commonly applied in the DII/E-DII literature, particularly in studies using NHANES data^(7,11,13)^. Second, our study described differences in the E-DII on a continuous scale. Many studies categorise the DII/E-DII scores into quartiles or percentiles, which has implications for the loss of information on variability and usefulness in moving the field forward to interventions. For example, the categorisation of the DII/E-DII scores into quartiles does not tell us what level of change in the DII/E-DII score is necessary to reduce the risk of a specific health outcome. This information could help inform the interventions needed to test the impact of the DII/E-DII scores on health outcomes.

The magnitude of socio-demographic differences in the E-DII scores was modest but generally reflected the patterns from other dietary index studies. We found that only 34 % of American adults reported a 24-hour diet intake associated with anti-inflammatory potential. This corroborates the findings of other studies suggesting that most American adults fall short of a healthy diet recommended by the U.S. Dietary Guidelines for Americans based on various dietary assessment measures^(4,42,43)^. Similar to studies assessing diet quality using the healthy eating index, alternate healthy eating index, Mediterranean or dietary approaches to stop hypertension, our study reported the highest anti-inflammatory dietary potential among females, older adults, Hispanics and those with high education levels or income^(4,43)^. Nonetheless, the absolute differences in the E-DII scores by the socio-demographic factors were modest. The E-DII scores in this study ranged from −5·96 (anti-inflammatory) to 4·90 (pro-inflammatory), but the average differences between the socio-demographic groups were < 1·0. Previous studies have observed an impact of a 1-point increase in DII score on a 6 % increased risk of depression, 8 % increased risk of CVD, 7 % increased risk of colorectal cancer and 8 % increased risk of overall mortality^(44–46)^. Unfortunately, the DII is not adjusted for overall energy intake; therefore, it is difficult to separate the independent effect of the inflammatory potential. Nevertheless, some prospective studies have found that a 1-point increase in the E-DII score is associated with a 5 % increased risk of all-cause mortality and 16 % increased odds of developing frailty^(46–48)^.

Randomised trials are needed to test the magnitude of change required in the E-DII score to reduce the risk of various adverse health outcomes^(26,49)^. Furthermore, long-term prospective studies are needed to test whether diets with anti-inflammatory E-DII scores confer greater health benefits than other dietary indices. Additionally, potential physiological differences may exist in the effect of an inflammatory diet on health outcomes, considering the presence of other inflammation-inducing factors, such as obesity, financial adversity, racism and discrimination^(2,3)^. Therefore, future studies should investigate potential subgroup differences to clarify the heterogeneity in the associations between the DII/E-DII scores and health outcomes^(7,11,50)^.

### Conclusions

Our cross-sectional study of 34 547 American adults compared two different approaches for handling missing data to investigate the socio-demographic differences in the E-DII scores. We found similar patterns of associations with each approach, although MI facilitated the inclusion of an additional 44 % of participants who were ineligible for CCA due to missing data. Based on the E-DII scores, > 50 % of American adults reported 24-hour diet intakes associated with pro-inflammatory potential, with modest differences by sex, age, race/ethnicity, education and income.
